# The Effect of Ranibizumab Loading Treatment on Vision-Related Quality of Life in Diabetic Macular Edema †

**DOI:** 10.3390/clinpract11030081

**Published:** 2021-09-14

**Authors:** Hatice Daldal, Mustafa Turkyilmaz, Melike Balikoglu Yilmaz, Ufuk Berberoglu

**Affiliations:** 1 Faculty of Medicine, Usak University, 64000 Usak, Turkey; ufuk.berberoglu@usak.edu.tr; 2 Tarsus State Hospital, 33460 Mersin, Turkey; drturkyilmaz@gmail.com; 3 Faculty of Medicine, Izmir Katip Celebi University, 35620 İzmir, Turkey; drmelkebalkoglu@yahoo.com

**Keywords:** diabetic macular edema, best-corrected visual acuity, intravitreal ranibizumab, central macular thickness, national eye institute 25-item visual function questionnaire

## Abstract

Aims: To investigate the changes in vision-related quality of life after a loading dose of three consecutive intravitreal ranibizumab (IVR) injections in patients with unilateral diabetic macular edema (DME). Materials and Methods: Fifty-two eyes of 52 patients who received IVR injections in only one eye with DME were included in our study. The following characteristics of the patients were recorded: gender, education status, marital status, work status, presence of chronic disease. The changes in best-corrected visual acuity (BCVA) and central macular thickness (CMT) were evaluated at baseline (before treatment) and 1 month after the third intravitreal injection (after treatment). Patients were administered the Turkish form of the National Eye Institute 25-Item Visual Functions Questionnaire (NEI VFQ-25 TR). The quality of life scores assessed by the NEI VFQ-25 TR, the BCVA, intraocular pressure (IOP), and CMT measurements were compared at baseline (before treatment) and 1 month after the third intravitreal injection (after treatment). Results: We enrolled 52 patients (25 females, 27 males) in our study; mean age was 64.35 ± 9.26 years. After treatment, BCVA improved significantly (*p* = 0.001), and macular thickness decreased significantly (*p* < 0.001). All NEI VFQ-25 TR subscale scores were significantly higher after treatment (*p* < 0.05). However, no significant correlation was found between the change in BCVA and CMT and the change in NEI VFQ-25 TR subscale and composite scores. The increase in near activities scores was significantly higher in males (*p* = 0.020) and in the retired group (*p* = 0.022). There were no significant differences in the changes in NEI VFQ-25 TR subscale and composite scores in relation to educational status. Discussion: Significant improvements in BCVA, macular edema, and vision-related quality of life were found in DME patients who received IVR injections with a loading dose, as shown by the NEI VFQ-25 TR. Interestingly, a significant improvement in quality of life was observed even though the patients could see well with the fellow eye. In conclusion, the NEI VFQ-25 TR is a useful scale to evaluate the changes in visual function and psychosocial characteristics of DME patients after treatment.

## 1. Introduction

Diabetic retinopathy and macular edema are important microvascular complications of diabetes mellitus. They are major causes of blindness in the working-age population in developed countries [1,2]. The social consequences of loss of function and vision are treatment costs and loss of labor [3]. This is due to the loss of central vision that occurs when the central retina and/or macula are affected [4]. It is estimated that there will be 366 million diabetic patients worldwide by 2030 [5].

The pathogenesis of diabetic macular edema (DME) is multifactorial and includes angiogenic, inflammatory, hypoxic, and hemodynamic processes leading to blood–retinal barrier disruption and leakage of intraretinal fluid [6,7,8]. Since its introduction into clinical use, intravitreal antivascular endothelial growth factor (anti-VEGF) has been the first-line treatment for DME [9].

Although methods such as best-corrected visual acuity (BCVA), fundus fluorescein angiography, and optical coherence tomography are used in the evaluation of treated patients, it has become apparent over time that the evaluation of the patient’s visual function is not sufficient and that quality of life studies should also be performed [3,10,11]. Quality of life studies not only provide information about the patient’s perspective, but also ensure that the most impaired function is considered in treatment planning [3]. The American National Eye Institute 25-Item Visual Functions Questionnaire (NEI VFQ-25) is a commonly used questionnaire to measure the effectiveness of treatments for many diseases [12].

The purpose of this study is to compare the vision-related quality of life of patients who received intravitreal ranibizumab (IVR) therapy for unilateral DME and have good vision in the fellow eye, at baseline (before treatment) and 1 month after the third intravitreal injection (after a loading therapy of three consecutive intravitreal injections).

## 2. Materials and Methods

Fifty-two consecutive patients with DME who presented to the ophthalmology clinic between February 2018 and July 2018 were included in our study.

The inclusion criteria were as follows: patients with unilateral DME who were naive to anti-VEGF injection or laser photocoagulation treatment due to peripheral neovascularization. Macular edema was identified according to the Early Treatment Diabetic Retinopathy Study classification. Clinically significant DME was defined as any of the following 3 criteria: (1) Retinal thickening within 500 microns of the foveal center. (2) Hard exudates within 500 microns of the foveal center that have adjacent retinal thickening. (3) Retinal thickening in at least one disk area, a portion of which is within one disk diameter of the foveal center [13]. The fellow eye of the patients did not require injection treatment according to the ETDRS.

The exclusion criteria were the following: patients with pathologies more likely to affect visual acuity than DME, such as macular degeneration, corneal opacity, glaucoma, uveitis, or retinal vein occlusion, patients who had undergone cataract surgery, Nd: YAG laser capsulotomy, or pan-retinal laser photocoagulation in the last six months. Patients with bilateral macular edema were also excluded to avoid biased selection.

The following patient characteristics were recorded: gender (female, male), education status (none, primary school, high school, university), marital status (married, not married), work status (working, non-working, retired), presence of chronic disease.

Intravitreal injections of 0.05 mL ranibizumab (Lucentis; Genentech USA Inc, San Francisco, CA/Novartis Ophthalmics, Basel, Switzerland) were administered three times monthly as a loading dose in all eyes. The injections were performed by the same physician (HD) in an operating room under sterile conditions.

Before and after the injections, the patients underwent a detailed ophthalmologic examination. Best-corrected visual acuity was assessed with the Snellen chart, intraocular pressure (IOP) was measured with Goldmann applanation tonometry, and anterior segment and fundus examinations were performed with a 90D lens. Central macular thickness (CMT) was measured with spectral-domain optical coherence tomography (OCT). Before treatment, patients underwent fundus fluorescein angiography (FFA).

BCVA, IOP, and CMT measurements and the Turkish form of the NEI VFQ-25 (NEI VFQ-25 TR) questionnaire were performed and the BCVA, IOP, CMT, and quality of life scores were compared at baseline (before treatment) and 1 month after the third injection (after treatment). Patients who did not have reading difficulties completed the questionnaire themselves; patients who could not read adequately due to vision problems were interviewed with the help of a relative.

This questionnaire was developed by Mangione et al. in 1998 [12]. The Turkish version of the NEI VFQ-25 questionnaire was also conducted, and its validity and reliability were studied by Ahmet Baris Toprak et al. as NEI VFQ-25 TR [10]. It consists of 38 questions, 25 of which were related to general health and vision, difficulties related to activities, and consequences of vision problems, and another 13 questions are related to subscales (general health, general vision, near vision, far vision, social function, driving, role restriction, well-being/distress, and addiction).

All statistical analyses were performed using SPSS v21 (SPSS Inc., Chicago, IL, USA). A Kolmogorov–Smirnov test was used to determine whether variables were normally distributed. Data are given as mean ± standard deviation and median (minimum–maximum) for continuous variables and frequency (percentage) for categorical variables. Repeated measures were evaluated using the Wilcoxon signed ranks test. For comparisons between groups, differences between results were analyzed using the Mann–Whitney U test or the Kruskal–Wallis test, depending on the number of groups. Spearman correlation coefficients were calculated to assess relationships between continuous variables. Two-tailed *p*-values of less than 0.05 were considered statistically significant.

## 3. Results

We enrolled 52 patients (25 females, 27 males) in our study; mean age was 64.35 ± 9.26 years (range 46–87 years). The summary of patient characteristics is shown in Table 1.

Best-corrected visual acuity (*p* = 0.001) improved significantly (1.00 ± 0.27 logMAR vs. 0.54 ± 0.20 logMAR) and macular thickness (*p* < 0.001) decreased significantly after treatment (401.79 ± 69.10 vs. 283.35 ± 39.41). On the other hand, IOP increased significantly after treatment (*p* = 0.019) and was within the normal range (12.42 ± 2.33 vs. 13.13 ± 2.09 (Table 2).

All NEI VFQ-25 TR subscale scores (general health (*p* = 0.001), general vision (*p* < 0.001), ocular pain (*p* < 0.001), near activities (*p* < 0.001), distance activities (*p* < 0.001), vision-specific social functioning (*p* = 0.002), vision-specific mental health (*p* < 0.001), vision-specific role difficulties (*p* < 0.001), vision-specific dependency (*p* < 0.001), driving (*p* = 0.010), color vision (*p* = 0.046), peripheral vision (*p* = 0.014)) and composite score (*p* < 0.001) were significantly higher after treatment (Table 3).

When assessing changes in the NEI VFQ-25 TR subscale and composite scores in relation to gender, we found an increase in the near activities scores, which were significantly higher in males than females (*p* = 0.020). There were no significant differences between genders in terms of changes in other NEI VFQ-25 TR subscale and composite scores (Table 4).

We found no significant differences between groups in changes in NEI VFQ-25 TR subscale and composite scores in relation to education status (Table 5).

Assessment of the changes in NEI VFQ-25 TR subscale and composite scores in relation to work status revealed that the increase in near activities scores was significantly higher in the retired group than in the non-working group (*p* = 0.022). In the working group, the increase in near activities scores was similar to the other groups. In addition, there were no significant differences between groups in changes in other NEI VFQ-25 TR subscale and composite scores (Table 6).

Looking at the changes in NEI VFQ-25 TR subscale and composite scores according to the presence of chronic disease (hypertension in twenty patients, coronary artery disease in three patients, chronic renal failure in one patient, chronic obstructive pulmonary disease in two patients), peripheral vision scores were detected to be significantly higher in patients without chronic disease (*p* = 0.022). There were no significant differences between groups in changes in other NEI VFQ-25 TR subscale and composite scores (Table 7).

We examined the correlations of age, BCVA before injection (at baseline), change in BCVA, macular thickness before injection (at baseline), and change in macular thickness with the changes in NEI VFQ-25 TR subscale and composite scores. We found no significant correlation between these variables and the NEI VFQ-25 TR subscale and composite scores (Table 8).

## 4. Discussion

Diabetic retinopathy is the most common cause of vision loss in developed countries for the 40- to 65-year-old age group [14]. The major cause of vision loss in diabetic patients is DME [15].

The baseline treatment options for DME are strict glycemic control and laser photo-coagulation treatment [16,17,18,19]. In some DME patients, treatments such as steroid and anti-VEGF injections, protein kinase C inhibitors, implantation of a corticosteroid-releasing intravitreal device, and pars plana vitrectomy have been used when laser photocoagulation was not sufficient [20].

VEGF is a proangiogenic cytokine mainly responsible for neovascularization in diabetic retinopathy [21]. In addition to its ability to increase vascular permeability, VEGF is a mitogenic, chemotactic, proinflammatory, and neuroprotective factor. It plays a role in the development of pathological angiogenesis under conditions leading to ocular ischemia. Retinal neovascularization is stimulated by VEGF released in response to retinal ischemia [22].

According to the International Council of Opthalmology (ICO) 2017 Guidelines for Diabetic Eye Care, DME is divided into two forms: with or without center impairment. Anti-VEGF treatment is recommended for patients with central DME who have visual acuity of 20/30 or worse [23]. Based on the guidelines, three anti-VEGF drugs (bevacizumab, ranibizumab, aflibercept) and a slow-release corticosteroid implants are available for first-line treatment of DME [24,25,26].

Ranibizumab is an antibody fragment against human VEGF-A. It is produced by *E. coli* using recombinant DNA technology. It inhibits all isoforms of VEGF-A and VEGF165, VEGF121, and VEGF110 [27]. The safety and efficacy of IVR have been evaluated in prospective, multicenter clinical trials [28,29].

Visual acuity is important to measure visual function, but it does not provide us with sufficient information about how patients’ lives are affected when they lose function. Visual acuity alone cannot measure post-injection recovery, changes in daily activities, visual satisfaction, visual impairment, depression, or loss of social function. In this case, another method of measurement is needed. Surveys are useful in this regard [30].

In Granström et al.’s study of 58 patients, vision (NEI VFQ-25) and general health (SF-36) questionnaires were used in patients receiving anti-VEGF treatment. Significant improvement in visual acuity and macular thickness was observed. For the subscales of general health, general vision, near activities, and mental health, and the composite score, the study found significant improvement within a short period of time from baseline to four months. General health, general vision, and near and distance activities improved from baseline to one year [31]. In our study, all NEI VFQ-25 TR subscale scores and the composite score improved significantly (with higher scores) after loading treatment. Granström et al. investigated the effect of anti-VEGF medication on vision-related and health-related quality of life [31]. In our study, we investigated the effect of ranibizumab, one of the anti-VEGF agents, on vision-related quality of life.

In the multicenter, 12-month, laser-assisted phase III of the RESTORE study of 345 patients, the NEI VFQ-25 composite score and vision-related subscales improved significantly from baseline with IVR alone and in combination with laser versus laser [32].

The RIDE and RISE studies by Bressler et al. examined the effect of IVR in patients with central DME using the NEI VFQ-25 questionnaire. Participants were divided into three groups: ranibizumab 0.3 mg, ranibizumab 0.5 mg, and sham treatment. The NEI VFQ-25 questionnaire was administered at baseline and at 6, 12, 18, and 24 months. They observed that IVR improved vision-related function and that the change in NEI VFQ-25 composite score was greater in the ranibizumab 0.3 mg- and 0.5 mg-treated group than in the sham treatment at 12 and 24 months, regardless of whether the better or worse seeing eye was treated [33].

Another study using data from the RIDE and RISE studies was conducted to examine how the NEI VFQ-25 responds to DME and to determine the change in the NEI VFQ-25 associated with a change in BCVA of ≥15 letters. The largest mean increases in NEI VFQ-25 subscale scores were found in patients with visual acuity gains of ≥15 letters. In RIDE, the mean changes in NEI VFQ-25 scores were +9.0, +14.8, +9.7, and +9.7, respectively, for the composite score, the near and distance activities subscales, and the vision dependency subscale; in RISE, they were +7.1, +12.6, +7.3, and +5.7, respectively. The mean change in the composite score and the distance and vision-specific dependency subscale scores was lower in patients who had lost ≥15 letters (RIDE: −6.6, −4.5, and −4.8, respectively, and RISE: −2.7, −5.8, and −1.7, respectively). The near activities subscale score increased slightly in this group of patients (RIDE: +1.9; RISE: +1.2). Overall, a BCVA gain of ≥15 letters by month 24 corresponded to an improvement in the NEI VFQ-25 composite score of ∼7 and 9 points in the RIDE and RISE studies, respectively. Conversely, patients who lost ≥15 letters of BCVA by month 24 experienced a decrease in the NEI VFQ-25 composite score of ~3 points to 6.5 points. This study confirms that the NEI VFQ-25 is sensitive to changes in BCVA over time in patients with DME [34].

In the RELIGHT study, which included 109 patients with DME receiving IVR injections, the correlations between BCVA in the study eye and the status of the eye at baseline (as better or worse after BCVA) and the NEI VFQ and, additionally, the Macular Disease Society Treatment Satisfaction Questionnaire (MacTSQ), were evaluated. The best-corrected visual acuity of the study eye correlated strongly with the NEI VFQ composite scores and most subscales, but not with the MacTSQ subscales. Statistically significant improvements were observed in most NEI VFQ subscales at 6, 12, and 18 months. For the MacTSQ, improvements between baseline scores and the scores at months 6, 12, and 18 were observed for subscale 1, but were statistically significant only at month 12. The lack of correlation between the BCVA and the MacTSQ suggests the presence of psychophysical factors that cannot be measured by conventional means [35].

Our study assessed quality of life differently with the NEI VFQ-25 TR questionnaire before and after IVR injections in patients with central DME in only one eye (with worse vision). Interestingly, s significant improvement in quality of life was found even though patients had good vision in the fellow eye.

However, in our study, we observed no significant differences between the groups following the assessment of changes in the NEI VFQ-25 TR subscale and composite scores in relation to educational status.

Using the NEI VFQ-25 TR questionnaire, our study concluded a significant improvement in quality of life-related visual function in patients with central DME who received IVR injections in a loading dose. Best-corrected visual acuity (*p* = 0.001) improved significantly, and macular thickness (*p* < 0.001) decreased significantly, after treatment. All NEI VFQ-25 TR subscale scores were significantly higher after treatment. However, no significant correlation was found between the change in VA, the CMT, and NEI VFQ-25 TR subscale and composite scores.

Assessment of the changes in NEI VFQ-25 TR subscale and composite scores in relation to gender revealed that the score for near activities was significantly higher in males than in females.

When assessing changes in the NEI VFQ-25 TR subscale and composite scores in relation to work status, an increase in near activities was detected in the retired group compared to the working and non-working groups. In the working group, the increase in near activities scores was similar to the other groups. In addition, there were no significant differences between groups in changes in other NEI VFQ-25 TR subscale and composite scores.

When changes in the NEI VFQ-25 TR subscale and composite scores were assessed in relation to the presence of chronic disease, there was a significant increase in peripheral vision scores in patients without chronic disease.

The limitations of the study are the short follow-up period and the small number of patients included. Further studies with more patients and longer follow-up are needed to evaluate the effect of education status, marital status, work status, and presence of chronic disease on questionnaire results and the validity of the questionnaire.

## 5. Conclusions

In conclusion, BCVA and macular edema improved significantly with treatment. Although it is an effective treatment method, we can only understand that it meets patients’ expectations and makes their lives easier with this questionnaire.

According to the NEI VFQ-25 TR questionnaire, significant improvement in vision-related quality of life was observed in DME patients who received IVR injections with a loading dose. In conclusion, this study has shown that the NEI VFQ-25 TR questionnaire is a useful scale to evaluate the changes in visual function, psychosocial characteristics, and vision-related quality of life of DME patients before and after injection, even when the macular edema is unilateral and the fellow eye has good vision.

## Figures and Tables

**Table 1:** Summary of patient characteristics.

Age	64.35 ± 9.26 (46–87)
Gender	
Female	25 (48.08%)
Male	27 (51.92%)
Education Status	
None	1 (1.92%)
Primary School	35 (67.31%)
High School	14 (26.92%)
University	2 (3.85%)
Married	52 (100.00%)
Work Status	
Working	12 (23.08%)
Non-working	25 (48.08%)
Retired	15 (28.85%)
Presence of Chronic Disease	23 (44.23%)

Data are given as mean ± standard deviation (minimum–maximum) for continuous variables and as frequency (percentage) for categorical variables.

**Table 2:** Summary of ocular findings before (baseline) and after treatment (1 month after the third injection).

Ocular Findings		Mean ± Standard Deviation (SD)	Median (Min–Max)	*p*
Best-Corrected Visual Acuity (log MAR) (*n* = 52)	Before	1.00 ± 0.27	1 (0.4–1.3)	0.001
	After	0.54 ± 0.20	0.52 (0.15–1)	
Intraocular Pressure (*n* = 52)	Before	12.42 ± 2.33	12 (9–16)	0.019
	After	13.13 ± 2.09	14 (8–17)	
Macular Thickness (*n* = 52)	Before	401.79 ± 69.10	387.5 (302–616)	<0.001
	After	283.35 ± 39.41	277.5 (212–417)	

**Table 3:** Summary of NEI VFQ-25 TR subscale and composite scores before (baseline) and after treatment (1 month after the third injection).

NEI VFQ-25 TR Scores		Mean ± Standard Deviation (SD)	Median (Min–Max)	*p*
General Health (*n* = 52)	Before	60.29 ± 15.16	65 (27.5–82.5)	0.001
	After	62.93 ± 13.16	65 (32.5–82.5)	
General Vision (*n* = 52)	Before	37.69 ± 14.68	37.5 (10–65)	<0.001
	After	56.54 ± 11.51	60 (37.5–82.5)	
Ocular Pain (*n* = 52)	Before	80.77 ± 16.51	81.25 (37.5–100)	<0.001
	After	84.38 ± 13.98	87.5 (50–100)	
Near Activities (*n* = 52)	Before	59.54 ± 19.50	62.5 (12.5–100)	<0.001
	After	69.31 ± 17.47	75 (12.5–100)	
Distance Activities (*n* = 52)	Before	68.43 ± 18.57	70.83 (25–100)	<0.001
	After	74.04 ± 15.71	77.09 (37.5–100)	
Vision-Specific Social Functioning (*n* = 52)	Before	80.29 ± 18.53	83.33 (33.33–100)	0.002
	After	82.69 ± 15.64	83.33 (41.67–100)	
Vision-Specific Mental Health (*n* = 52)	Before	73.37 ± 19.14	80 (20–100)	<0.001
	After	78.65 ± 17.15	80 (25–100)	
Vision-Specific Role Difficulties (*n* = 52)	Before	62.62 ± 20.69	68.75 (6.25–93.75)	<0.001
	After	70.79 ± 19.44	75 (6.25–100)	
Vision-Specific Dependency (*n* = 52)	Before	74.04 ± 23.98	81.25 (0–100)	<0.001
	After	78.49 ± 22.90	87.5 (0–100)	
Driving (*n* = 24)	Before	43.06 ± 33.75	54.17 (0–83.33)	0.010
	After	47.22 ± 35.75	66.67 (0–83.33)	
Color Vision (*n* = 52)	Before	86.06 ± 18.13	100 (25–100)	0.046
	After	87.98 ± 16.78	100 (25–100)	
Peripheral Vision (*n* = 52)	Before	72.12 ± 20.20	75 (25–100)	0.014
	After	75.00 ± 17.85	75 (25–100)	
Composite (*n* = 52)	Before	68.28 ± 15.65	72.13 (27.88–94.63)	<0.001
	After	74.51 ± 14.24	77.59 (30.13–97)	

**Table 4:** Summary of the changes in NEI VFQ-25 TR subscale and composite scores after treatment (1 month after the third injection) according to gender.

NEI VFQ-25 TR Scores	Gender	*N*	Mean ± Standard Deviation (SD)	Median (Min–Max)	*p*
General Health	Female	25	2.30 ± 5.40	0 (0–22.5)	0.477
	Male	27	2.96 ± 5.68	0 (−5–17.5)	
General Vision	Female	25	18.00 ± 10.23	17.5 (−7.5–40)	0.655
	Male	27	19.63 ± 10.53	17.5 (5–40)	
Ocular Pain	Female	25	3.50 ± 5.73	0 (0–12.5)	0.934
	Male	27	3.70 ± 6.77	0 (0–25)	
Near Activities	Female	25	7.17 ± 5.96	8.33 (0–20.83)	0.020
	Male	27	12.19 ± 8.41	12.5 (0–33.33)	
Distance Activities	Female	25	5.17 ± 4.70	4.17 (0–16.67)	0.521
	Male	27	6.02 ± 5.34	4.17 (0–20.83)	
Vision-Specific Social Functioning	Female	25	2.00 ± 4.36	0 (0–16.67)	0.611
	Male	27	2.78 ± 6.11	0 (−8.33–25)	
Vision-Specific Mental Health	Female	25	5.20 ± 6.20	5 (0–25)	0.780
	Male	27	5.37 ± 5.53	5 (0–20)	
Vision-Specific Role Difficulties	Female	25	9.75 ± 7.67	12.5 (0–25)	0.111
	Male	27	6.71 ± 8.65	6.25 (−6.25–31.25)	
Vision-Specific Dependency	Female	25	3.25 ± 6.02	0 (−6.25–18.75)	0.218
	Male	27	5.56 ± 7.42	0 (0–31.25)	
Driving	Female	3	2.78 ± 4.81	0 (0–8.33)	0.834
	Male	21	4.37 ± 6.78	0 (0–16.67)	
Color Vision	Female	25	1.00 ± 5.00	0 (0–25)	0.341
	Male	27	2.78 ± 8.01	0 (0–25)	
Peripheral Vision	Female	25	3.00 ± 8.29	0 (0–25)	0.921
	Male	27	2.78 ± 8.01	0 (0–25)	
Composite	Female	25	5.79 ± 3.12	5.33 (0.5–13.08)	0.431
	Male	27	6.62 ± 3.59	5.61 (2.61–18.33)	

Negative values represent a decrease in the selected score after treatment (1 month after the third injection).

**Table 5:** Summary of the changes in NEI VFQ-25 TR subscale and composite scores after treatment (1 month after the third injection) according to education status.

NEI VFQ-25 TR Scores	Education Status	*n*	Mean ± Standard Deviation (SD)	Median (Min–Max)	*p*
General Health	Primary school or less	36	2.71 ± 5.68	0 (−5–22.5)	0.960
	High school or higher	16	2.50 ± 5.24	0 (0–17.5)	
General Vision	Primary school or less	36	17.78 ± 9.46	17.5 (−7.5–40)	0.272
	High school or higher	16	21.25 ± 12.01	22.5 (5–40)	
Ocular Pain	Primary school or less	36	4.51 ± 6.78	0 (0–25)	0.117
	High school or higher	16	1.56 ± 4.27	0 (0–12.5)	
Near Activities	Primary school or less	36	8.91 ± 7.19	8.33 (0–29.17)	0.209
	High school or higher	16	11.72 ± 8.64	10.42 (0–33.33)	
Distance Activities	Primary school or less	36	5.56 ± 5.27	4.17 (0–20.83)	0.732
	High school or higher	16	5.73 ± 4.53	4.17 (0–16.67)	
Vision-Specific Social Functioning	Primary school or less	36	2.55 ± 5.92	0 (−8.33–25)	0.959
	High school or higher	16	2.08 ± 3.73	0 (0–8.33)	
Vision-Specific Mental Health	Primary school or less	36	5.56 ± 6.41	5 (0–25)	0.933
	High school or higher	16	4.69 ± 4.27	5 (0–15)	
Vision-Specific Role Difficulties	Primary school or less	36	7.99 ± 7.56	6.25 (−6.25–25)	0.894
	High school or higher	16	8.59 ± 9.92	6.25 (0–31.25)	
Vision-Specific Dependency	Primary school or less	36	3.65 ± 5.86	0 (−6.25–18.75)	0.301
	High school or higher	16	6.25 ± 8.54	3.13 (0–31.25)	
Driving	Primary school or less	13	5.77 ± 7.12	0 (0–16.67)	0.165
	High school or higher	11	2.27 ± 5.39	0 (0–16.67)	
Color Vision	Primary school or less	36	2.08 ± 7.01	0 (0–25)	0.797
	High school or higher	16	1.56 ± 6.25	0 (0–25)	
Peripheral Vision	Primary school or less	36	2.78 ± 7.97	0 (0–25)	0.886
	High school or higher	16	3.13 ± 8.54	0 (0–25)	
Composite	Primary school or less	36	6.14 ± 3.17	5.42 (0.5–14.38)	0.992
	High school or higher	16	6.41 ± 3.89	5.94 (2.42–18.33)	

Negative values represent a decrease in the selected score after treatment (1 month after the third injection).

**Table 6:** Summary of the changes in NEI VFQ-25 TR subscale and composite scores after treatment (1 month after the third injection) according to working status.

NEI VFQ-25 TR Scores	Working Status	*n*	Mean ± Standard Deviation (SD)	Median (Min–Max)	*p*
General Health	Working	12	3.33 ± 5.87	0 (0–17.5)	0.531
	Non-working	25	3.00 ± 5.73	0 (0–22.5)	
	Retired	15	1.50 ± 4.98	0 (−5–17.5)	
General Vision	Working	12	20.21 ± 11.84	20 (5–35)	0.838
	Non-working	25	18.50 ± 9.87	17.5 (−7.5–40)	
	Retired	15	18.33 ± 10.42	17.5 (5–40)	
Ocular Pain	Working	12	1.04 ± 3.61	0 (0–12.5)	0.227
	Non-working	25	4.50 ± 6.12	0 (0–12.5)	
	Retired	15	4.17 ± 7.72	0 (0–25)	
Near Activities	Working	12	11.46 ± 7.35	10.42 (0–25)	0.022
	Non-working	25	6.83 ± 5.50	8.33 (0–20.83)	
	Retired	15	13.33 ± 9.48	12.5 (0–33.33)	
Distance Activities	Working	12	5.56 ± 3.70	4.17 (0–12.5)	0.623
	Non-working	25	5.00 ± 4.96	4.17 (0–16.67)	
	Retired	15	6.67 ± 6.06	4.17 (0–20.83)	
Vision-Specific Social Functioning	Working	12	2.08 ± 3.77	0 (0–8.33)	0.910
	Non-working	25	2.00 ± 4.98	0 (−8.33–16.67)	
	Retired	15	3.33 ± 6.90	0 (0–25)	
Vision-Specific Mental Health	Working	12	4.58 ± 4.50	5 (0–15)	0.810
	Non-working	25	5.00 ± 5.95	5 (0–25)	
	Retired	15	6.33 ± 6.67	5 (0–20)	
Vision-Specific Role Difficulties	Working	12	8.33 ± 7.69	9.38 (0–25)	0.334
	Non-working	25	9.25 ± 8.29	12.5 (−6.25–25)	
	Retired	15	6.25 ± 8.84	6.25 (0–31.25)	
Vision-Specific Dependency	Working	12	5.73 ± 6.23	3.13 (0–12.5)	0.595
	Non-working	25	3.75 ± 6.25	0 (−6.25–18.75)	
	Retired	15	4.58 ± 8.34	0 (0–31.25)	
Driving	Working	10	3.33 ± 5.83	0 (0–16.67)	0.473
	Non-working	5	1.67 ± 3.73	0 (0–8.33)	
	Retired	9	6.48 ± 8.1	0 (0–16.67)	
Color Vision	Working	12	2.08 ± 7.22	0 (0–25)	0.566
	Non-working	25	1.00 ± 5.00	0 (0–25)	
	Retired	15	3.33 ± 8.80	0 (0–25)	
Peripheral Vision	Working	12	4.17 ± 9.73	0 (0–25)	0.722
	Non-working	25	3.00 ± 8.29	0 (0–25)	
	Retired	15	1.67 ± 6.45	0 (0–25)	
Composite	Working	12	6.25 ± 2.31	6.99 (2.42–9.47)	0.723
	Non-working	25	5.82 ± 3.17	5.25 (0.5–13.08)	
	Retired	15	6.87 ± 4.38	5.61 (2.8–18.33)	

Negative values represent a decrease in the selected score after treatment (1 month after the third injection).

**Table 7:** Summary of the changes in NEI VFQ-25 TR subscale and composite scores after treatment (1 month after the third injection) according to the presence of chronic disease.

NEI VFQ-25 TR Scores	Chronic Disease	*n*	Mean ± Standard Deviation (SD)	Median (Min–Max)	*p*
General Health	Absent	29	2.67 ± 5.78	0 (0–22.5)	0.872
	Present	23	2.61 ± 5.25	0 (−5–17.5)	
General Vision	Absent	29	20.43 ± 11.02	22.5 (−7.5–40)	0.120
	Present	23	16.85 ± 9.21	17.5 (5–40)	
Ocular Pain	Absent	29	2.59 ± 5.15	0 (0–12.5)	0.232
	Present	23	4.89 ± 7.29	0 (0–25)	
Near Activities	Absent	29	8.62 ± 6.09	8.33 (0–25)	0.397
	Present	23	11.23 ± 9.27	8.33 (0–33.33)	
Distance Activities	Absent	29	5.46 ± 4.18	4.17 (0–16.67)	0.946
	Present	23	5.80 ± 5.99	4.17 (0–20.83)	
Vision-Specific Social Functioning	Absent	29	2.59 ± 4.51	0 (0–16.67)	0.506
	Present	23	2.17 ± 6.26	0 (−8.33–25)	
Vision-Specific Mental Health	Absent	29	5.17 ± 5.90	5 (0–25)	0.838
	Present	23	5.43 ± 5.82	5 (0–20)	
Vision-Specific Role Difficulties	Absent	29	9.05 ± 8.11	6.25 (0–25)	0.350
	Present	23	7.07 ± 8.49	6.25 (−6.25–31.25)	
Vision-Specific Dependency	Absent	29	4.09 ± 6.31	0 (−6.25–18.75)	0.764
	Present	23	4.89 ± 7.53	0 (0–31.25)	
Driving	Absent	15	2.78 ± 5.14	0 (0–16.67)	0.253
	Present	9	6.48 ± 8.10	0 (0–16.67)	
Color Vision	Absent	29	2.59 ± 7.75	0 (0–25)	0.425
	Present	23	1.09 ± 5.21	0 (0–25)	
Peripheral Vision	Absent	29	5.17 ± 10.31	0 (0–25)	0.022
	Present	23	0.00 ± 0.00	0 (0–0)	
Composite	Absent	29	6.41 ± 2.93	5.88 (0.5–13.08)	0.210
	Present	23	5.98 ± 3.91	4.96 (2.25–18.33)	

Negative values represent a decrease in the selected score after treatment (1 month after the third injection).

**Table 8:** The correlations of different variables with the changes in NEI VFQ-25 TR subscale and composite scores.

NEI VFQ-25 TR Scores		Age	Best-Corrected Visual Acuity (logMAR) (Before)	Best-Corrected Visual Acuity (logMAR) (Change)	Macular Thickness (Before)	Macular Thickness (Change)
Age	*r*	-	0.030	−0.094	0.014	−0.057
	*p*	-	0.831	0.507	0.920	0.688
Changes in NEI VFQ-25 TR Subscale and Composite Scores						
General Health	*r*	−0.192	−0.002	−0.006	0.179	0.102
	*p*	0.173	0.988	0.966	0.205	0.471
General Vision	*r*	0.209	0.020	0.071	−0.102	−0.072
	*p*	0.137	0.889	0.615	0.473	0.612
Ocular Pain	*r*	−0.118	−0.024	0.167	−0.028	−0.036
	*p*	0.406	0.868	0.238	0.843	0.798
Near Activities	*r*	−0.037	−0.213	−0.101	−0.193	−0.019
	*p*	0.796	0.130	0.475	0.170	0.891
Distance Activities	*r*	−0.022	0.177	0.122	−0.100	−0.157
	*p*	0.879	0.208	0.387	0.482	0.266
Vision-Specific Social Functioning	*r*	0.125	0.014	0.036	0.027	−0.009
	*p*	0.376	0.922	0.800	0.850	0.951
Vision-Specific Mental Health	*r*	−0.035	0.144	0.055	0.098	0.090
	*p*	0.808	0.307	0.697	0.490	0.526
Vision-Specific Role Difficulties	*r*	−0.049	0.057	0.136	−0.063	−0.051
	*p*	0.732	0.690	0.336	0.659	0.718
Vision-Specific Dependency	*r*	−0.205	−0.185	−0.099	−0.049	0.100
	*p*	0.145	0.189	0.486	0.732	0.480
Driving	*r*	0.214	0.155	0.320	0.300	0.398
	*p*	0.315	0.470	0.128	0.154	0.054
Color Vision	*r*	−0.014	−0.106	−0.080	−0.048	−0.070
	*p*	0.919	0.453	0.574	0.735	0.623
Peripheral Vision	*r*	−0.086	−0.101	−0.056	−0.175	−0.140
	*p*	0.543	0.474	0.692	0.216	0.321
Composite	*r*	−0.056	−0.049	0.075	−0.139	−0.062
	*p*	0.691	0.730	0.596	0.325	0.663
